# The clinicopathological and prognostic value of CD44 expression in bladder cancer: a study based on meta-analysis and TCGA data

**DOI:** 10.1080/21655979.2020.1765500

**Published:** 2020-05-20

**Authors:** Yu Hu, Yongrui Zhang, Jialin Gao, Xin Lian, Yuantao Wang

**Affiliations:** a Department of Pathology, China-Japan Union Hospital, Jilin University, Changchun, Jilin, China; b Department of Urology, The First Hospital of Jilin University, Changchun, Jilin, China

**Keywords:** Survival, lymph node metastasis, expression, CD44, bladder cancer

## Abstract

CD44 is reported to be involved in tumor invasion and metastasis. However, the role of cancer stem cell marker CD44 in bladder cancer still remains controversial. Hence, the correlations between CD44 expression and the clinicopathological features and the prognosis of bladder cancer were investigated. Publications using immunohistochemical methods were identified. The Cancer Genome Atlas (TCGA) data were also analyzed. The odds ratios (ORs) or hazard ratios (HRs) with their 95% confidence intervals (95% CIs) were calculated. 14 studies involving 1107 tissue samples were included. CD44 expression in bladder cancer was lower than in non-tumor tissue samples (OR = 0.14, P = 0.005), which was consistent with TCGA data. CD44 expression was correlated with advanced T stage (OR = 1.76, P = 0.029) and lymph node metastasis (OR = 4.09, P < 0.001). Multivariate survival analysis showed that CD44 expression was not linked to tumor-specific survival, overall survival, and recurrence/relapse-free survival, but was associated with disease failure (HR = 2.912, 95% CI = 1.51–5.61). No relationships of CD44 expression with the clinicopathological features and overall survival were found from TCGA data. Our finding suggested that CD44 expression may be correlated with progression, metastasis, and disease failure of bladder cancer. However, further large-scale studies are needed.

**Abbreviations**: CD44: Cluster of Differentiation 44; CIs: Confidence Intervals; CSCs: Cancer Stem Cells; EMT: Epithelial-mesenchymal Transition; HRs: Hazard Ratios; ORs: Odds Ratios; TCGA: The Cancer Genome Atlas

## Graphical Abstract

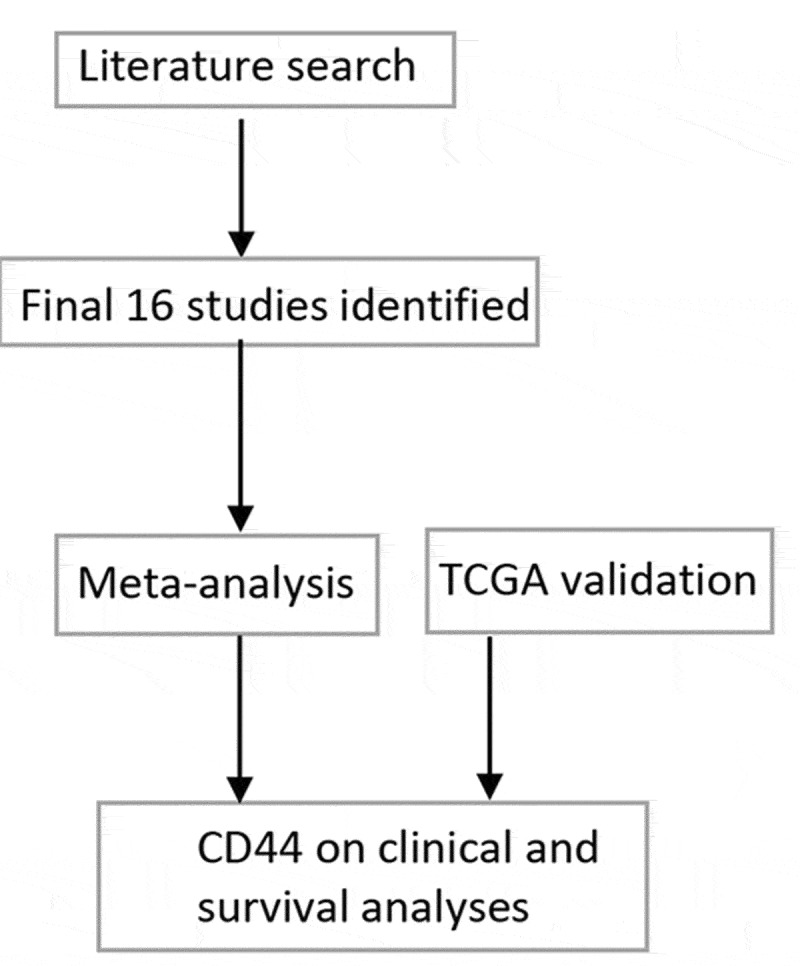

## Background

Bladder cancer is one of the most common malignancy of the urinary system in the world [1]. According to global cancer statistics, approximately 549,393 new cases will be diagnosed with bladder cancer across the world 2018, with approximately 199,922 cases died from bladder cancer [1]. Although improvements in current therapeutic methods such as surgery, radiation therapy, and chemotherapy have shown a better clinical outcome for early-stage patients, advanced stage patients with bladder cancer have a much worse prognosis and the estimated 5-year survival rate remains at 5%-35% [2–4]. Therefore, it is needed to find a novel biomarker as an effective therapeutic target for improving the prognosis of patients with bladder cancer.

Cancer stem cells (CSCs), a small subpopulation within tumor cells, are responsible for self-renewal, uncontrolled proliferation and differentiation [5,6]. Increasing evidence suggests that CSCs are associated with cancer progression, metastasis, recurrence, and drug resistance [7,8]. Cluster of differentiation 44 (CD44), a complex transmembrane glycoprotein, is a receptor for hyaluronan and is located on chromosome 11p13 [9,10]. CD44 is involved in many important functions such as cell growth, survival, differentiation, and motility, cell-cell adhesion, the regulation of epithelial-mesenchymal transition (EMT), apoptosis resistance, tumor cell metastasis and invasion [11–14]. CD44 has also been implicated as a CSC marker in human cancers [15]. CD44 plays a crucial role in tumor progression, metastasis, and chemoresistance [16]. Studies on CD44 expression have been identified in various cancers, such as ovarian cancer, breast cancer, and oral cancer etc [17–20]. CD44 expression is associated with worse prognosis in gastric cancer [21], head and neck squamous cell carcinoma [22], and osteosarcoma [23]. However, CD44 expression is linked to favorable prognosis in prostate cancer [24]. Some studies have reported that CD44 is frequently expressed in bladder cancer [25,26].

However, the clinical role of CD44 expression in patients with bladder cancer is still controversial. For example, CD44 expression was associated with tumor grade by Lipponen 1998 et al [27]. No relationship was found between CD44 expression and tumor grade by Gadalla 2004 et al [28]. Therefore, the current study was performed to investigate the relationships of CD44 expression with the clinicopathological features and the prognosis in patients with bladder cancer.

## Materials and methods

### Literature search strategy

A comprehensive literature search was performed in the electronic databases (PubMed, EMBASE, EBSCO, Web of Science, and the Cochrane Library databases) prior to 30 December 2018. The following search terms and key words were applied to identify eligible publications: ‘CD44 OR cluster of differentiation 44ʹ, ‘expression’, ‘bladder OR urothelial’, ‘cancer OR carcinoma OR tumor OR neoplasm’. The reference lists of the eligible studies were also manually screened to determine other relevant articles.

### Selection criteria

Studies were included if they met the following selection criteria: 1) patients were diagnosed with bladder cancer; 2) studies used immunohistochemical (IHC) detection of pan-CD44/CD44s expression in tissue samples; 3) studies evaluated the relationship between CD44 expression and the clinicopathological parameters; 4) studies assessed the association of CD44 expression between bladder cancer and control groups; 5) studies provided sufficient information to estimate the prognosis of CD44 expression on patients with bladder cancer using multivariate survival analysis. For the studies with duplicate sample data, only the last or the most complete study was selected. The main exclusion criteria were as follows: 1) reviews, abstracts, cell or animal studies; 2) literature published using duplicate data; 3) insufficient data.

### Data extraction

Two independent authors extracted the following data, any disagreements were resolved by discussion among all the authors. The following information was recorded: first author’s surname, year of publication, sample source, median or mean age, cancer stage, antibody source, cutoff value, detection method, number of cases and controls, frequency of expression, clinicopathological features, and survival information of multivariate analysis.

### TCGA dataset

Clinical information for bladder cancer was downloaded from The Cancer Genome Atlas (TCGA) data portal. Finally, 406 cases with the available clinical information and corresponding RNA sequencing data were included. 19 normal tissue samples were also included.

### Statistical analysis

Meta-analysis was performed using Stata software 12.0 (StataCorp LP, College Station, TX, USA). The association of CD44 expression between bladder cancer and control samples was evaluated using the pooled odds ratios (ORs) and the corresponding 95% confidence intervals (95% CIs). The pooled ORs with 95% CIs were also applied to assess the correlations between CD44 expression and the clinicopathological features. The overall hazard ratios (HRs) with 95% CIs were calculated to evaluate the prognostic role of CD44 expression on patients with bladder cancer. Heterogeneity between studies was measured by using the Cochran’s Q statistic [29]. The random-effects model (DerSimonian and Laird method) was applied in this study. When a substantial heterogeneity was measured (*P* < 0.1), sensitivity analyses were conducted to estimate the stability of the re-calculated results by removing one study [30,31]. Potential publication bias was detected by using Egger’s linear regression test if more than nine studies were included [32].

For TCGA data, the difference in CD44 expression between bladder cancer and non-tumor tissue samples was measured using the t-test. CD44 expression was divided into low and high expression group according to the median value. The univariate logistic regression analysis was performed to analyze the relationships between CD44 expression and the clinical features. Survival curve was plotted by Kaplan–Meier method with log-rank test. The univariate and multivariate Cox hazards regression models were applied to analyze the impact of CD44 expression on the prognosis if possible. Data were analyzed using R (v. 3.5.1, Institute for Statistics and Mathematics, Vienna, Austria).

## Results

### Study characteristics

**Figure 1.**
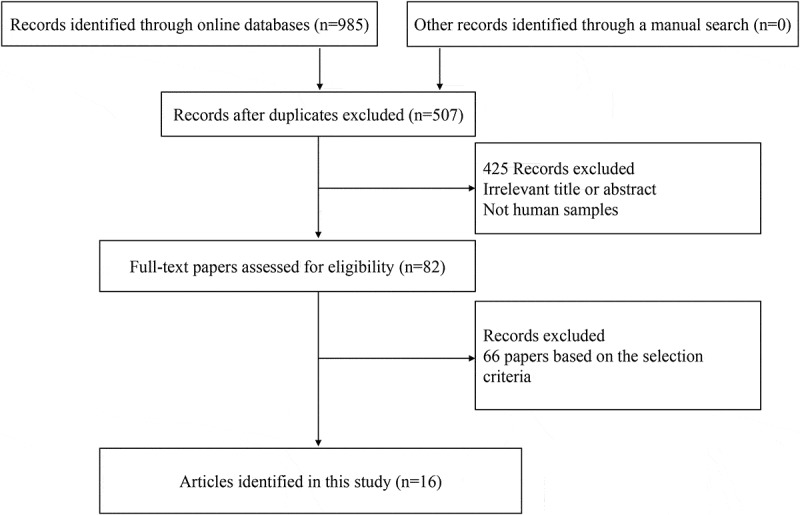
Flow diagram of the study selection.

Figure 1 shows the detailed procedure of the article screening process. Based on the above inclusion criteria, finally, 14 studies, including 1038 cases with bladder cancer and 69 non-tumor controls, were deemed eligible for CD44 expression using IHC methods [25–28,33-42]. All eligible studies were published between 1996 and 2018. Among these eligible studies, five studies analyzed the association between bladder cancer and non-tumor tissues [28,38–40,42]. Ten studies involving 829 patients with bladder cancer assessed the relationships of CD44 expression with clinicopathological characteristics [25–28,33,34,36,39–41]. Four studies involving 448 cases recorded the prognostic information using multivariate survival analysis [27,33,35,37]. The basic characteristics of the included studies are shown in Table 1.

**Table 1.** The basic characteristics of the eligible studies.

First author	Country	Age	Stage	Antibodies	Positivity (IHC)	Control Sample	Cancer	Control	Grade 3–4	Grade 1–2	pT2-4	pTa-1	Lymph node metastasis (yes)	Lymph node metastasis (no)	Clinical outcome-MA
							N (E+ %)	N (E+ %)	E+/N	E+/N	E+/N	E+/N	E+/N	E+/N	
Sugino 1996 ^26^	UK	NA	pTa-pT4	Hermes 3	NA		37 (73%)		12/22	15/15	9/19	18/18			No
Woodman 1996 ^42^	UK	NA	NA	Hermes 3	NA	Normal	19 (100%)	5 (100%)							No
Müller 1997 ^41^	Germany	NA	pTa-pT4	F10-44-2, Boehringer Mannheim, Germany, No. 1441272	>30%		35 (100%)		14/14	21/21	8/8	27/27			No
Lipponen 1998 ^27^	Finland	67	pTa-pT4	clone 2C5, R&D Systems, Abingdon, U.K.	Moderate-strong		170 (28.2%)		15/33	32/137	13/36	35/134	10/21	38/149	Yes
Kong 2003 ^40^	China	NA	pTa-pT4	Ab-2,Oncogene Science, Uniondale, NY	NA	Non-cancerous	56 (28.6%)	28 (67.8%)			5/29	11/25			No
Gadalla 2004 ^28^	Egypt	NA	pTa-pT4	Mob104, B734, Clone A020, Isotype IgG2b, Diagnostic BioSystems, New Delhi, India	Weak-strong	Normal	55 (67.3%)	8 (87.5%)	11/22	16/22	23/40	14/15			No
Kuncová 2007 ^25^	Czech Republic	NA	pTa-pT4	DAKO Glostrup, Denmark	Moderate-strong		122 (86.9%)		25/34	81/88					No
Omran 2012 ^39^	Egypt	NA	pT1-pT3	Dako, Denmark	Weak-strong	Normal	50 (80%)	11 (90.9%)	3/6	12/14	31/37	9/13			No
Oliva 2013 ^38^	USA	NA	CIS	Pharmingen, San Diego, CA	Scores 2–3	Non-cancerous	17 (5.9%)	17 (88.2%)							No
Hofner 2014 ^37^	Germany	65	pT1-pT4	NA	>75%		107 (NA)								Yes
Afonso 2015 ^36^	Portugal	70	pTa-pT4	AbD Serotec, MCA2726	Scores ≥4		114 (50%)				38/68	19/46			No
Koukourakis 2016 ^35^	Greece	NA	pT2-pT3	Ab6124, Abcam, Cambridge, UK	>5%		66 (42.4%)								Yes
Wu 2017 ^34^	China	NA	pT2-pT4	Cell Signaling Technology, Danvers	IRS ≥2		85 (52.9%)						15/18	30/67	No
Wu 2018 ^33^	China	NA	pT2-pT4	NA	IRS ≥2		105 (52.4%)						21/26	34/79	Yes

N: number of the study population; E+: positive expression; MA: multivariate analysis; NA: not applicable; IRS: immunoreactive score; IHC: immunohistochemistry.

### CD44 expression in bladder cancer and non-tumor tissues

**Figure 2.**
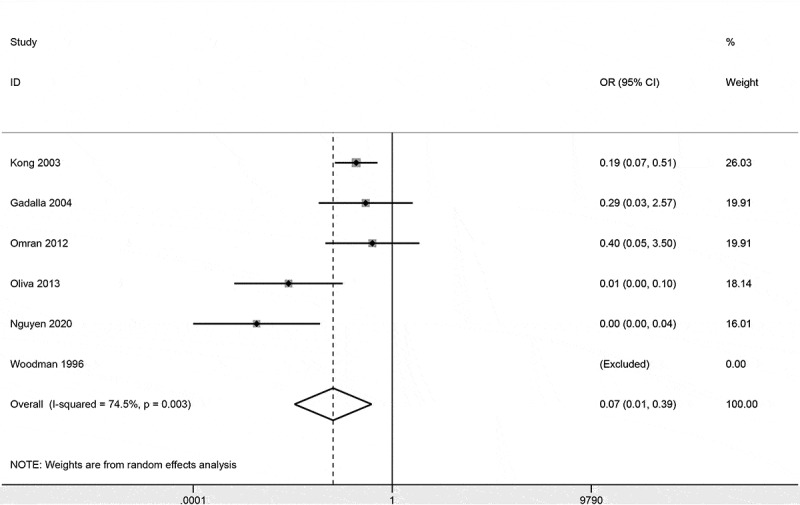
Forest plot of the association of CD44 expression between bladder cancer and non-tumor tissue samples.

Data from five studies included 197 patients with bladder cancer and 69 non-tumor tissue samples, which showed that CD44 expression was lower in bladder cancer than in non-tumor tissue samples (OR = 0.14, 95% CI = 0.04–0.54, *P* = 0.005) (Figure 2).

### Relationship between CD44 expression and clinicopathological characteristics

**Figure 3.**
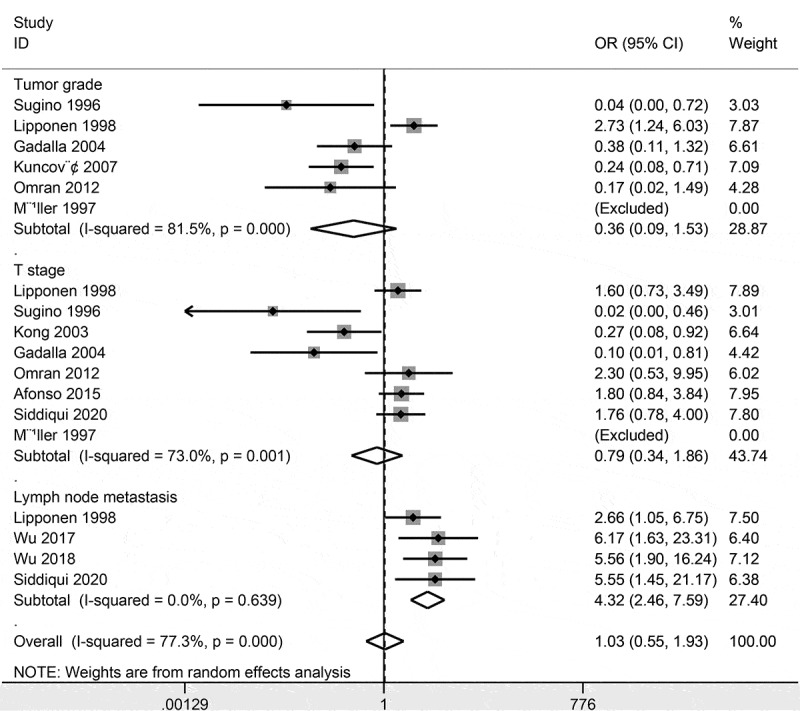
Forest plot of the association of CD44 expression with the clinicopathological characteristics.

Data involving six studies with 428 cases demonstrated no association between CD44 expression and tumor grade (OR = 0.36, 95% CI = 0.09–1.53, *P* = 0.167) (Figure 3). CD44 expression was not correlated with T stage (OR = 0.62, 95% CI = 0.21–1.79, *P* = 0.374) (Figure 3), including seven studies with 515 cases.

Data involving three studies with 360 bladder cancer patients showed that CD44 expression was significantly correlated with lymph node metastasis (OR = 4.09, 95% CI = 2.20–7.62, *P* < 0.001) (Figure 3).

### Sensitivity analyses

Slight heterogeneity was found in bladder cancer and non-tumor controls (*P* = 0.09), when the study of Oliva 2013 et al [38] was removed, the re-calculated OR was 0.23 (95% CI = 0.10–0.52, *P* < 0.001), with no heterogeneity (*P* = 0.798). Significant heterogeneity was found between CD44 expression and tumor grade (*P* < 0.001) and T stage (*P* = 0.001). When we removed the study of Lipponen 1998 et al [27] and re-calculated the pooled result regarding the correlation between CD44 expression and tumor grade (OR = 0.24, 95% CI = 0.11–0.50, *P* < 0.001), resulting in a significantly decreased heterogeneity (*P* = 0.535). We successively removed these three studies – Sugino 1996 et al [26], Kong 2003 et al [40], and Gadalla 2004 et al [28], and re-calculated the pooled OR between CD44 expression and T stage (OR = 1.76, 95% CI = 1.06–2.93, *P* = 0.029), with no obvious evidence of heterogeneity (*P* = 0.910).

### Prognostic role of CD44 expression using multivariate survival analysis

**Figure 4.**
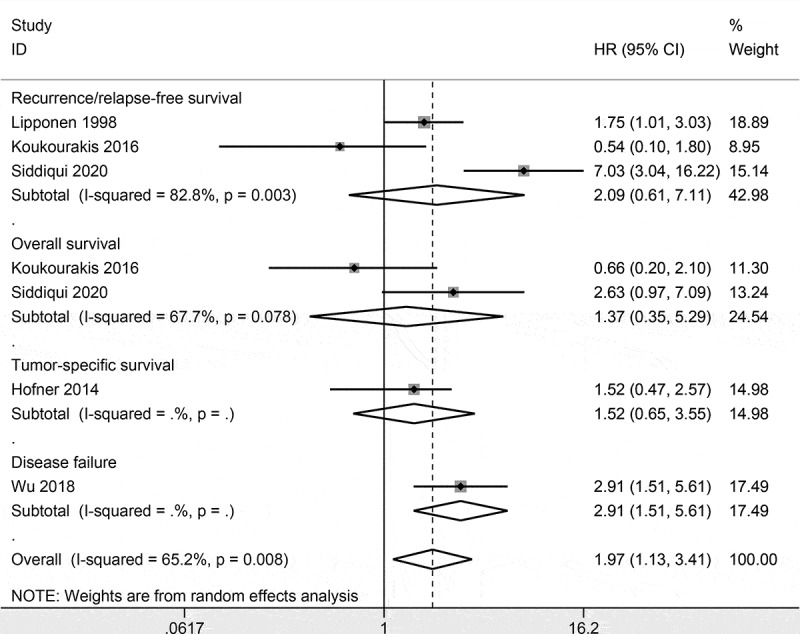
Forest plot of the prognostic role of CD44 expression using multivariate survival analysis.

CD44 expression was reported to be not correlated with tumor-specific survival in 107 patients (HR = 1.52, 95% CI = 0.47–2.57) [37] and be not associated with overall survival (HR = 0.66, 95% CI = 0.2–2.1) [35] in 66 cases. The pooled data from two studies with 236 cases showed no association between CD44 expression and recurrence/relapse-free survival (HR = 1.18, 95% CI = 0.40–3.51, *P* = 0.759) (Figure 4). CD44 expression was significantly correlated with disease failure (HR = 2.912, 95% CI = 1.51–5.61) [33] in 105 patients.

### TCGA

**Figure 5.**
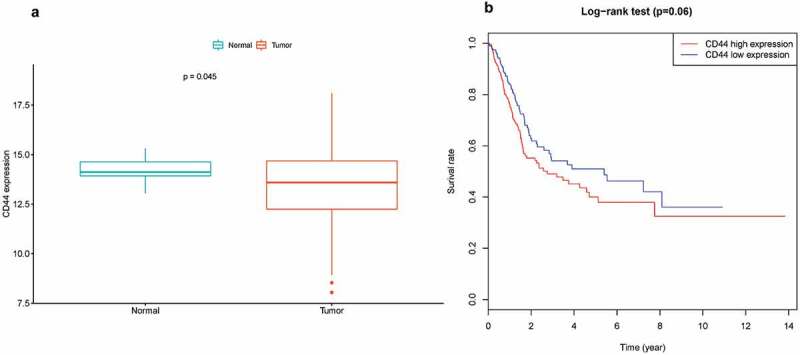
Association between CD44 expression and bladder cancer, (a) expression level of CD44 between bladder cancer and normal tissue samples; (b) Kaplan-Meier survival analysis of CD44 expression in bladder cancer.

Expression level of CD44 in bladder cancer was lower than in normal tissues (*P* = 0.045) (Figure 5(a)).

Univariate analysis using logistic regression demonstrated that CD44 high expression was not correlated with age, gender, tumor grade, T stage, lymph node metastasis, and distal metastasis (all *P* values > 0.05) (Table 2).

**Table 2.** Association of CD44 expression with the clinicopathological characteristics from TCGA data.

Factors	Total (N)	OR with 95% CI	*P*
Age (≥69 vs. <69 years)	406	0.94 (0.64–1.39)	0.766
Gender (Male vs. female)	406	1.08 (0.69–1.68)	0.735
Grade (High vs. low)	403	1.01 (0.41–2.47)	0.991
T stage (T3-4 vs. T1-2)	373	1.01 (0.65–1.55)	0.978
Lymph node metastasis (Positive vs. negative)	364	0.86 (0.56–1.32)	0.495
Metastasis (Positive vs. negative)	206	0.65 (0.19–2.3)	0.508

N: number of the study population; OR: odds ratio; 95% CI: 95% confidence interval; TCGA: The Cancer Genome Atlas.

Kaplan-Meier survival analysis was performed, which revealed that CD44 expression was not linked to overall survival (*P* = 0.06) (Figure 5(b)).

## Discussion

CSCs possess self-renewal and high tumor-initiating ability, which cause the progression, metastasis and recurrence of cancer. Thus, eliminating CSCs are critical for cancer therapy [43,44]. Extensive evidence has suggests that CD44 plays crucial roles in tumor aggressiveness and metastasis, especially with CSCs related characteristics [45]. CD44 participates in important biological events in the invasion process and epithelial mesenchymal transition (EMT) [46]. CD44 is also shown to be associated with treatment resistance and CD44 expression is correlated with poor survival of many types of human cancers [47]. Loss of CD44 expression is associated with poor prognosis in prostate cancer [24]. CD44 is highly expressed across a wide variety of human cancers including bladder cancer [25,48]. Although many studies have reported the role of CD44 expression in bladder cancer. The clinical effect of CD44 expression in patients with bladder cancer has not been clearly investigated. The current study was first carried out to explore the clinical significance of CD44 expression and its expression on the survival of patients with bladder cancer.

Pooled data showed that CD44 expression was significantly lower in bladder cancer than in non-tumor tissue samples. Slight heterogeneity was detected, when we deleted the study of Oliva 2013 et al [38], heterogeneity was lacking and the re-calculated OR remained significant, which suggested that our analysis was stable and credible. Moreover, further TCGA data demonstrated that expression level of CD44 in bladder cancer was also lower than in normal tissue samples, which was further confirmed from TCGA dataset.

We further analyzed whether CD44 expression was linked to the clinicopathological characteristics of patients with bladder cancer. CD44 expression using IHC method was not linked to tumor grade and T stage, but was closely associated with lymph node metastasis. Substantial heterogeneity was measured between CD44 expression and tumor grade and T stage. Sensitivity analyses were further conducted. We removed the study of Lipponen 1998 et al [27] in relation to tumor grade and removed three studies of Sugino 1996 et al [26], Kong 2003 et al [40], and Gadalla 2004 et al [28] in relation to T stage. Based on sensitivity analyses, the re-calculated pooled results showed a negative correlation between CD44 expression and advanced tumor grade and a positive association between CD44 expression and advanced T stage. The possible factors and reasons might influence the pooled results, which were not clearly found. Because Lipponen 1998 et al [27] reported that CD44 expression was positively associated with advanced tumor grade. These three studies [26,28,40] reported that CD44 expression was negatively correlated with advanced T stage. Additionally, TCGA data demonstrated that CD44 high expression was not linked to tumor grade, T stage, lymph node metastasis, and distal metastasis. The above analyses revealed that CD44 expression may be associated with advanced T stage and lymph node metastasis based on IHC detection. However, the results may not be stable between CD44 expression and tumor grade and T stage. Additional studies are needed to further confirm these results in the future.

CD44 expression was not associated with tumor-specific survival [37], overall survival [35], and recurrence/relapse-free survival using multivariate analysis in bladder cancer. CD44 expression was reported to be significantly associated with disease failure from multivariate survival analysis [33], suggesting that CD44 may be a potential marker for predicting disease failure in bladder cancer. More studies with large sample sizes are essential to further validate the prognostic role of CD44 expression on patients with bladder cancer.

There were some limitations in this meta-analysis. First, the main ethnic population were European, and other ethnic groups, such as Asians and Africans, were not very sufficient. Second, the pooled analyses of the relationships between CD44 expression and the prognosis and patient clinicopathological features such as tumor grade and T stage. Additional studies are necessary to further obtain these reliable results. Third, we did not include unpublished articles and conference abstracts into meta-analysis because of insufficient information, which may lead to the selection bias.

## Conclusion

In conclusion, the current study showed that CD44 expression in bladder cancer was lower than in non-tumor tissue samples. CD44 expression may be correlated with tumor grade, advanced T stage and lymph node metastasis. CD44 expression was not correlated with tumor-specific survival, overall survival, and recurrence/relapse-free survival, but was associated with disease failure from multivariate survival analysis. Moreover, no association between CD44 expression and the clinicopathological features and overall survival was found from TCGA data. Additional large-scale prospective studies are essential to further validate our results in the future.

## Data Availability

The datasets used and/or analyzed during the current study are available from the corresponding author on reasonable request.
